# Integrated host/microbe metagenomics enables accurate lower respiratory tract infection diagnosis in critically ill children

**DOI:** 10.1172/JCI165904

**Published:** 2023-04-03

**Authors:** Eran Mick, Alexandra Tsitsiklis, Jack Kamm, Katrina L. Kalantar, Saharai Caldera, Amy Lyden, Michelle Tan, Angela M. Detweiler, Norma Neff, Christina M. Osborne, Kayla M. Williamson, Victoria Soesanto, Matthew Leroue, Aline B. Maddux, Eric A.F. Simões, Todd C. Carpenter, Brandie D. Wagner, Joseph L. DeRisi, Lilliam Ambroggio, Peter M. Mourani, Charles R. Langelier

**Affiliations:** 1 Chan Zuckerberg Biohub, San Francisco, California, USA.; 2 Division of Pulmonary, Critical Care, Allergy and Sleep Medicine, Department of Medicine, and; 3 Division of Infectious Diseases, Department of Medicine, University of California, San Francisco, San Francisco, California, USA.; 4 Chan Zuckerberg Initiative, San Francisco, California, USA.; 5 Department of Pediatrics, University of Colorado and Children’s Hospital Colorado, Aurora, Colorado, USA.; 6 Department of Biostatistics and Informatics, Colorado School of Public Health, University of Colorado, Aurora, Colorado, USA.; 7 Department of Biochemistry and Biophysics, University of California, San Francisco, San Francisco, California, USA.; 8 Department of Pediatrics, University of Arkansas for Medical Sciences and Arkansas Children’s Research Institute, Little Rock, Arkansas, USA.

**Keywords:** Infectious disease, Pulmonology, Bioinformatics, Expression profiling, Molecular diagnosis

## Abstract

**BACKGROUND:**

Lower respiratory tract infection (LRTI) is a leading cause of death in children worldwide. LRTI diagnosis is challenging because noninfectious respiratory illnesses appear clinically similar and because existing microbiologic tests are often falsely negative or detect incidentally carried microbes, resulting in antimicrobial overuse and adverse outcomes. Lower airway metagenomics has the potential to detect host and microbial signatures of LRTI. Whether it can be applied at scale and in a pediatric population to enable improved diagnosis and treatment remains unclear.

**METHODS:**

We used tracheal aspirate RNA-Seq to profile host gene expression and respiratory microbiota in 261 children with acute respiratory failure. We developed a gene expression classifier for LRTI by training on patients with an established diagnosis of LRTI (*n* = 117) or of noninfectious respiratory failure (*n* = 50). We then developed a classifier that integrates the host LRTI probability, abundance of respiratory viruses, and dominance in the lung microbiome of bacteria/fungi considered pathogenic by a rules-based algorithm.

**RESULTS:**

The host classifier achieved a median AUC of 0.967 by cross-validation, driven by activation markers of T cells, alveolar macrophages, and the interferon response. The integrated classifier achieved a median AUC of 0.986 and increased the confidence of patient classifications. When applied to patients with an uncertain diagnosis (*n* = 94), the integrated classifier indicated LRTI in 52% of cases and nominated likely causal pathogens in 98% of those.

**CONCLUSION:**

Lower airway metagenomics enables accurate LRTI diagnosis and pathogen identification in a heterogeneous cohort of critically ill children through integration of host, pathogen, and microbiome features.

**FUNDING:**

Support for this study was provided by the Eunice Kennedy Shriver National Institute of Child Health and Human Development and the National Heart, Lung, and Blood Institute (UG1HD083171, 1R01HL124103, UG1HD049983, UG01HD049934, UG1HD083170, UG1HD050096, UG1HD63108, UG1HD083116, UG1HD083166, UG1HD049981, K23HL138461, and 5R01HL155418) as well as by the Chan Zuckerberg Biohub.

## Introduction

Lower respiratory tract infection (LRTI) causes more deaths each year than any other type of infection and disproportionately affects children (1–4). The ability to accurately determine whether LRTI underlies or contributes to respiratory failure in the intensive care unit and to identify the etiologic pathogens is critical for effective and targeted treatments. However, LRTI diagnosis is challenging because noninfectious respiratory conditions can appear clinically similar. Moreover, no microbiologic diagnosis is obtained in many cases of suspected LRTI, since standard tests (such as bacterial culture) suffer from a narrow spectrum of targets and limited sensitivity (3, 5–8). At the same time, children are especially susceptible to false positive diagnoses due to frequent incidental carriage of potentially pathogenic microbes (3, 5, 9–12). As such, LRTI treatment is often empirical, leading to antimicrobial overuse, selection for resistant pathogens, and adverse outcomes (13–15).

Profiling host gene expression in the blood has shown promise as an innovative modality for diagnosing respiratory infection in hospitalized patients (16, 17). However, this approach has not been well studied in the diagnostically challenging critically ill pediatric population. Moreover, while blood gene expression can in some cases distinguish between the response to viral and bacterial infection (16–21), it cannot pinpoint the specific pathogens active in the respiratory tract, which is critical for optimal antimicrobial therapy.

Metagenomic next-generation sequencing (mNGS) of lower airway samples (e.g., tracheal aspirate [TA]) has the potential to detect pathogens and host gene expression signatures of LRTI (22). Whether such an approach can be successfully applied at scale for the purpose of clinical diagnosis remains unclear. Its applicability in a pediatric population has also never been examined despite well-established age-related differences in LRTI epidemiology (3, 9), rates of incidental pathogen carriage (3, 5, 9), and the immune response to infection (23, 24). Furthermore, to our knowledge, no metagenomic approach for LRTI diagnosis thus far integrates host and microbial features into a single diagnostic output, a crucial step toward streamlined clinical application.

Here, we performed metagenomic RNA-Seq of TA in a prospective cohort of 261 children with acute respiratory failure requiring mechanical ventilation. We developed a host gene expression classifier for LRTI by training on patients with an LRTI diagnosis supported by clinical microbiologic testing and patients with respiratory failure due to noninfectious causes. We then developed a classifier that integrates host, pathogen, and microbiome features to accurately diagnose LRTI and identify the likely causal pathogens, including in cases with negative clinical microbiologic testing. Our results demonstrate the feasibility of lower airway metagenomics for improved LRTI diagnosis in a large and heterogeneous cohort and reveal the importance of profiling both the pulmonary immune response and microbiome in a pediatric population.

## Results

### Patient cohort and LRTI adjudication.

**Figure 1:**
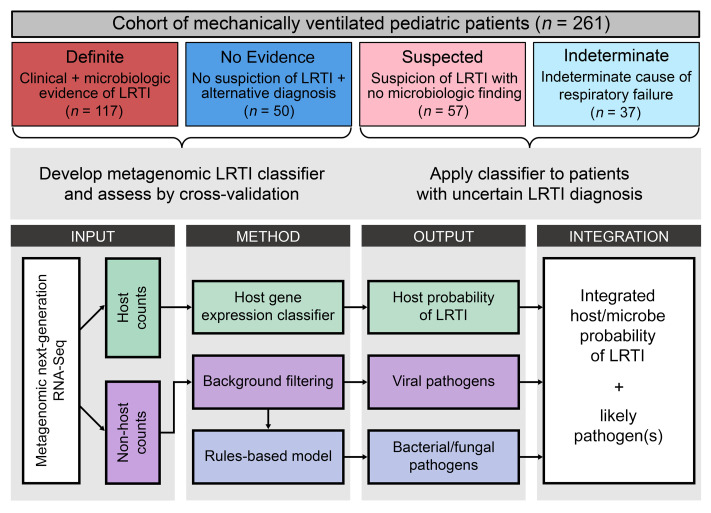
Study overview. Pediatric patients with acute respiratory failure requiring mechanical ventilation were clinically adjudicated into 4 LRTI status groups. The patients in the Definite and No Evidence groups, whose LRTI status was presumed to be known, were used to develop an integrated host/microbe mNGS classifier for LRTI and to evaluate its performance by cross-validation. The classifier was then applied to the patients in the Suspected and Indeterminate groups, whose LRTI status was considered uncertain. The integrated mNGS classifier takes into account a host probability of LRTI, derived from the host gene counts, and features of any viral or bacterial/fungal pathogens, derived from the nonhost (microbial) taxon counts.

We enrolled children with acute respiratory failure requiring mechanical ventilation at 8 hospitals in the United States between February 2015 and December 2017, as previously described (9, 25). TA was collected within 24 hours of intubation and underwent mNGS of RNA to assay host gene expression and detect respiratory microbiota (Figure 1). High-quality host gene expression and microbial data were obtained for 261 patients (Supplemental Data File 1).

Adjudication of LRTI status was performed without knowledge of the mNGS results and depended on the combination of two elements: (a) a retrospective clinical diagnosis made by study-site clinicians, who reviewed all clinical, laboratory, and imaging data available at the end of the admission, and (b) any standard-of-care respiratory microbiologic diagnostics (NP swab viral PCR and/or TA culture) performed on specimens collected during the first 48 hours of intubation. Patients were assigned to their LRTI status group as follows: (a) Definite, if clinicians made a diagnosis of LRTI and the patient had clinical microbiologic findings; (b) Suspected, if clinicians made a diagnosis of LRTI, but there were no microbiologic findings; (c) Indeterminate, if no diagnosis of LRTI was made despite some microbiologic findings; and (d) No Evidence, if clinicians identified a clear noninfectious cause of acute respiratory failure and no clinical or microbiologic suspicion of LRTI arose. We note that comprehensive microbiologic testing was not always performed in the No Evidence group in the absence of clinical suspicion.

The Definite and No Evidence groups were used to develop the metagenomic classifiers and to evaluate their performance by cross-validation due to the high degree of confidence in their clinical diagnoses (Figure 1). The patients in the Definite group were 39% female, with a median age of 0.5 years (IQR, 0.2–1.8), while the patients in the No Evidence group were 50% female, with a median age of 6.5 years (IQR, 1.5–12.9; Table 1 and Supplemental Figure 1; supplemental material available online with this article; https://doi.org/10.1172/JCI165904DS1). The difference in the age distribution of these groups (*P* < 0.001, Mann-Whitney test) reflected recognized epidemiological distinctions in the conditions that typically lead to respiratory failure in very young versus older children (3, 5).

Within the Definite group, 95% of patients were intubated by 2 days from hospital admission, indicative of community-acquired infection (Table 1). Clinical microbiologic testing identified viral infection alone in 46% of patients, bacterial infection alone in 14% of patients, and viral/bacterial coinfection in 40% of patients. The most common pathogens were respiratory syncytial virus (RSV) and *Haemophilus influenzae*, which frequently co-occurred (9). Diagnoses in the No Evidence group included trauma, neurological conditions, cardiovascular disease, airway abnormalities, ingestion of drugs/toxins, and sepsis that was clearly unconnected to LRTI. Nevertheless, most patients received antibiotic treatment by the time of TA sample collection in both the Definite (96%) and No Evidence (84%) groups (Table 1).

### Classification of LRTI status based on TA host gene expression features.

We first compared TA host gene expression between the Definite and No Evidence groups to determine whether it could distinguish patients based on LRTI status, regardless of the underlying cause of infection. We identified 4,718 differentially expressed genes at a Benjamini-Hochberg–adjusted *P* < 0.05 (Supplemental Figure 2A and Supplemental Data File 2). As expected, gene set enrichment analysis identified elevated expression of pathways involved in the immune response to infection in the Definite group (Supplemental Figure 2B and Supplemental Data File 3). Pathways related to the interferon response, a hallmark of antiviral innate immunity, were most strongly upregulated, consistent with the high prevalence of viral infections in the Definite group. Additional immune pathways upregulated in this group included Toll-like receptor signaling, cytokine signaling, inflammasome activation, neutrophil degranulation, antigen processing, and B cell and T cell receptor signaling. Conversely, pathways with reduced expression in the Definite group included translation, cilium assembly, and lipid metabolism (Supplemental Figure 2B and Supplemental Data File 3).

Because we observed a clear host signature of infection, we developed a classification approach to distinguish the patients in the Definite and No Evidence groups based on gene expression and evaluated its performance by 5-fold cross-validation. For each train/test split, we (a) used LASSO logistic regression on the samples in the training folds to select a parsimonious set of informative genes, (b) trained a random forest classifier using the selected genes, and (c) applied it to the samples in the test fold to obtain a host probability of LRTI.

**Figure 2:**
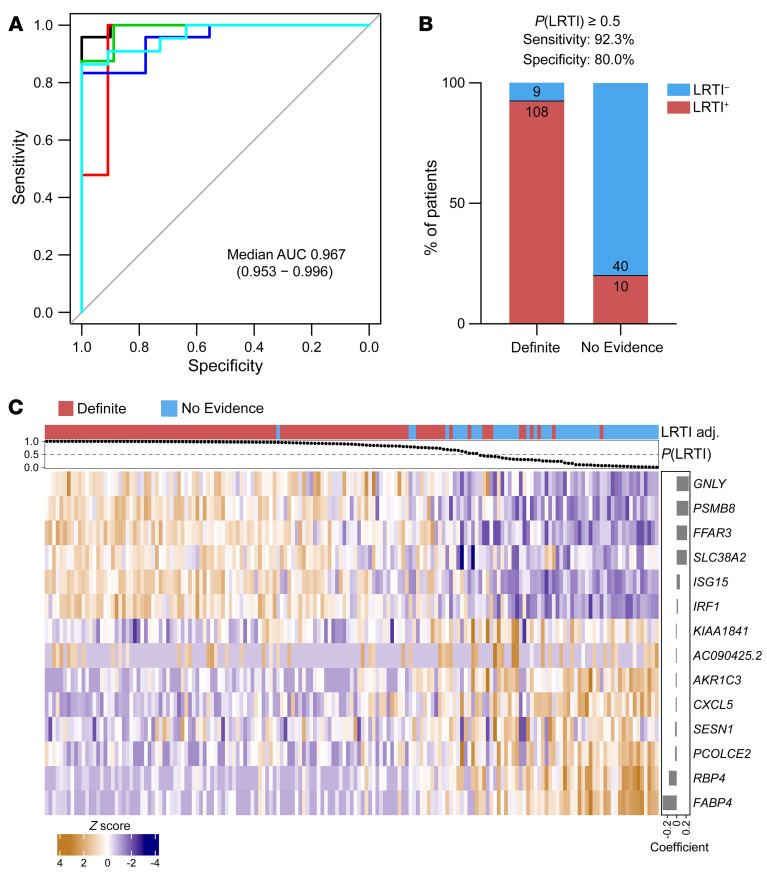
Host gene expression classifier for LRTI diagnosis. (**A**) Receiver operating characteristic (ROC) curve of the host gene expression classifier in each of the test folds. The median and range of the area under the curve (AUC) are indicated. (**B**) The number and percentage of patients in the Definite and No Evidence groups who were classified according to their clinical adjudication using a 50% out-of-fold probability threshold. (**C**) Heatmap showing standardized variance-stabilized expression values across all patients (columns) for the 14 final classifier genes (rows) selected from the full Definite and No Evidence data set. Shown are the LRTI adjudication (top colored horizontal bar) and out-of-fold LRTI probability (top dot plot) of each patient and the regression coefficient of each selected gene (side bar plot).

Our approach yielded a median area under the receiver operating characteristic curve (AUC) of 0.967 (range, 0.953–0.996), with the number of genes selected for use in the classifier ranging from 11 to 25 across the 5 train/test splits (Figure 2A and Supplemental Table 1). Using a 50% out-of-fold probability threshold to classify a patient as suffering from LRTI (LRTI^+^), the classifier assigned 92% of patients in the Definite group and 80% of patients in the No Evidence group according to their clinical LRTI adjudication (Figure 2B).

Having validated the performance of our approach by cross-validation, we then applied LASSO logistic regression to all the patients in the Definite and No Evidence groups to select a final set of genes (*n* = 14) for later classification of patients with Suspected or Indeterminate LRTI status (Figure 2C and Supplemental Table 2). As expected, the genes in the final classifier set that were assigned high absolute regression coefficients were also repeatedly selected in the cross-validation procedure (Supplemental Table 2).

The selected genes with the most positive regression coefficients, corresponding to higher expression in the Definite group, were *GNLY*, encoding an antibacterial peptide present in cytolytic granules of cytotoxic T cells and natural killer cells (26); *SLC38A2*, encoding a glutamine transporter upregulated in CD28-stimulated T cells (27, 28); *FFAR3*, encoding a G protein–coupled receptor activated by short-chain fatty acids that is induced by alveolar macrophages upon infection (29); and the interferon-stimulated genes *PSMB8*, *ISG15*, and *IRF1* (Figure 2C and Supplemental Table 2).

The selected genes with the most negative regression coefficients, corresponding to lower expression in the Definite group, were *FABP4*, encoding a fatty acid-binding protein considered a marker of alveolar macrophages, whose expression in the lung decreases in patients with LRTI, including COVID-19 (30–32), and *RBP4*, encoding a retinol-binding protein, whose expression in the lung also sharply decreases following onset of LRTI (30) and whose expression by macrophages in vitro is depressed by inflammatory stimuli (33) (Figure 2C and Supplemental Table 2).

We examined the expression of the final classifier genes as a function of patient age to confirm that their selection was not influenced by the different age distributions of the Definite and No Evidence groups (Supplemental Figure 3). Reassuringly, we found no significant difference in the expression of the 14 genes when comparing patients in the No Evidence group under the age of 4 (*n* = 23; median age, 1.3 years) and over the age of 4 (*n* = 27; median age, 12.5 years) (Supplemental Table 3A). Furthermore, we found that expression of 12 of the genes remained significantly different when comparing only children under the age of 4 in the Definite (*n* = 100; median age, 0.4 years) and No Evidence (*n* = 23; median age, 1.3 years) groups (Supplemental Table 3B).

### Detection of pathogens by mNGS and definition of microbial classification features.

We proceeded to analyze the microbial mNGS data to nominate likely pathogens whose features could be integrated into the LRTI classifier. We processed the TA samples alongside water controls through the Chan Zuckerberg ID (CZ-ID) metagenomic analysis pipeline (https://czid.org/) to obtain a count matrix of microbial taxa. The water controls allowed us to generate a background count distribution for each taxon, which modeled the contribution of contamination by microbes present in the laboratory environment or reagents.

**Figure 3.**
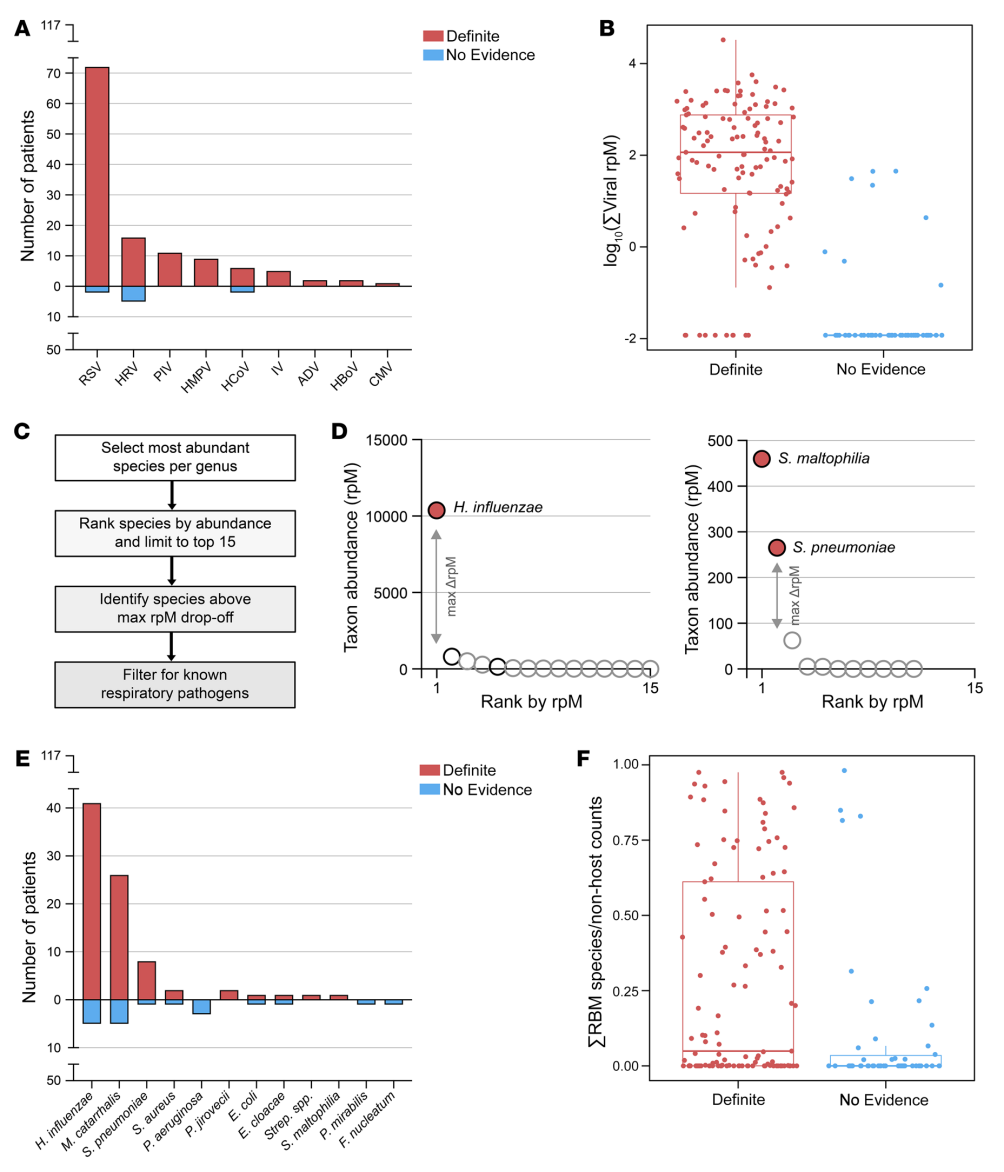
Metagenomic identification of respiratory pathogens. (**A**) Bar plot showing the distribution of viruses detected by mNGS after background filtering in patients in the Definite and No Evidence groups. RSV, respiratory syncytial virus; HRV, human rhinovirus; PIV, parainfluenza virus; HMPV, human metapneumovirus; HCoV, human coronavirus; IV, influenza virus; ADV, adenovirus; HBoV, human bocavirus; CMV, cytomegalovirus. (**B**) Box plot showing the log_10_-transformed summed abundance, measured in reads-per-million (rpM), of all pathogenic viruses detected in each patient, separated by group. Prior to log_10_-transformation, the minimum non-zero rpM value in the data set was divided by 10 and added to all the samples. Horizontal lines denote the median, box hinges represent the interquartile range (IQR), and whiskers extend to the most extreme value no greater than 1.5 × IQR from the hinges. (**C**) Analysis steps applied as part of the rules-based model (RBM), a heuristic approach designed to identify potential bacterial/fungal pathogens in the context of LRTI. (**D**) Graphical illustration of the RBM results in two representative patients from the Definite group. Each dot represents a bacterial/fungal species most abundant in its respective genus. Species above the maximum drop-off in rpM are colored in red; otherwise, the color is white. Species on the list of known respiratory pathogens have black outlines; otherwise, the outline is gray. (**E**) Bar plot showing the distribution of bacteria/fungi called as potential pathogens by the RBM in patients in the Definite and No Evidence groups. *Strep*. *spp.*, *Streptococcus* species other than *S*. *pneumoniae*. (**F**) Box plot showing the proportion of the RBM-identified pathogen(s) out of all nonhost counts in each patient, separated by group. Horizontal lines denote the median, box hinges represent the interquartile range (IQR), and whiskers extend to the most extreme value no greater than 1.5 × IQR from the hinges.

Viruses with known ability to cause LRTI that were present at an abundance statistically exceeding their background distribution were considered probable pathogens. By this criterion, we detected viruses in the lungs of 107 of 117 (91%) patients in the Definite group, with RSV being the most prevalent (Figure 3A). Among patients in the No Evidence group, 8 of 50 (16%) also had viruses detected by mNGS, which were probably missed clinically in the absence of characteristic symptoms. We defined the summed abundance of all pathogenic viruses detected in a patient, measured in reads-per-million (rpM), as the patient’s “viral score” for later use in an integrated host/microbe classifier (Figure 3B).

Because most patients in the Definite group had a positive NP swab viral PCR test, we could compare the viruses detected by PCR and mNGS (Supplemental Data File 4). The comparison was complicated, however, by the fact that PCR was performed on upper airway samples, so a virus detected by PCR was not necessarily present in the lower airway. Bearing this in mind, we found that 99 of 101 (98%) patients in the Definite group with a viral PCR hit also had a virus detected by mNGS, and both approaches detected at least 1 virus in common in 91 (92%) of those patients (Supplemental Figure 4A). Most cases in which NP swab PCR detected a virus, but mNGS did not, involved adenovirus (Supplemental Figure 4B). mNGS alone detected viruses in 8 of 16 (50%) patients in the Definite group lacking a viral PCR hit (Supplemental Figure 4A). We additionally performed viral PCR on the same TA samples subjected to mNGS in a subset of patients in the Definite group (*n* = 21), and 96% of PCR hits were detected by mNGS in this direct comparison (Supplemental Table 4).

Bacterial and fungal taxa in the mNGS data also underwent background filtering to retain only those present at an abundance statistically exceeding their background distribution based on water controls. Because incidental carriage of potentially pathogenic bacteria is common in children, we additionally applied a previously published algorithm to distinguish possible pathogens from commensals, called the rules-based model (RBM) (9, 22). The RBM identifies bacteria and fungi with known pathogenic potential that are relatively dominant in a sample (Figure 3, C and D), based on the principle that uncontrolled growth of a pathogen leads to reduced lung microbiome α-diversity in the context of LRTI (22, 34–36) (Supplemental Figure 4, C and D).

The RBM identified possible bacterial/fungal pathogens in 78 of 117 (66%) patients in the Definite group, with the most common being *H*. *influenzae*, *Moraxella catarrhalis*, and *Streptococcus pneumoniae* (Figure 3E). The RBM also identified potential bacterial/fungal pathogens in 17 of 50 (34%) patients in the No Evidence group. Patients in the Definite group with an RBM-identified pathogen exhibited markedly lower bacterial α-diversity compared with patients in the Definite group without an RBM-identified pathogen and compared with patients in the No Evidence group (Supplemental Figure 4D). In contrast, patients in the No Evidence group with an RBM-identified pathogen did not typically exhibit a loss of bacterial α-diversity (Supplemental Figure 4D), and in such cases, the RBM-identified species was far less dominant (Figure 3F). We, therefore, defined the patient’s “bacterial score” for use in an integrated host/microbe classifier as the proportion of the nonhost counts assigned to the RBM-identified pathogens, a measure of relative dominance (Figure 3F).

We next sought to compare the bacterial and fungal pathogens identified by mNGS with those found by culture of TA samples (Supplemental Data File 4). Importantly, mNGS can detect organisms that are challenging to grow in culture or are inhibited by previous antibiotic treatment, and the RBM selects the likeliest pathogen based on a global view of the microbiome. Despite these inherent differences between culture and the RBM, we found that in 44 of 63 (70%) patients in the Definite group who had a positive culture, at least 1 pathogen identified by the RBM was also found by culture (Supplemental Figure 4E). In the remaining 19 patients, the RBM identified a different species than culture (*n* = 7) or no pathogen at all (*n* = 12). Even in these cases, the species grown in culture was usually present in the mNGS data, but other species were more dominant (Supplemental Figure 4E). The RBM also identified a potential pathogen in 27 of 54 (50%) patients in the Definite group lacking a positive culture (Supplemental Figure 4E). Most cases where the species grown in culture was absent from the mNGS data after background filtering involved *Staphylococcus aureus*, *Streptococcus* species other than *S*. *pneumoniae*, and *E*. *coli* (Supplemental Figure 4F).

### Host gene expression differences between viral and bacterial LRTI.

Overall, mNGS identified viral and/or bacterial pathogens in 114 of 117 (97%) patients in the Definite group. Having established by mNGS which patients had an exclusively bacterial infection (*n* = 7), an exclusively viral infection (*n* = 36), or a viral/bacterial coinfection (*n* = 71), we went back and examined how effectively the top host classifier genes captured these different scenarios (Supplemental Figure 5A). As expected, some of the interferon-stimulated genes (e.g., *ISG15*) provided much more discriminating power for patients with a viral infection as compared with those with a purely bacterial infection. Reassuringly, however, several other classifier genes behaved similarly regardless of the underlying infection type.

We then asked more broadly whether host gene expression differed between patients with any bacterial LRTI (including viral coinfection) and patients with purely viral LRTI. We identified 108 differentially expressed genes at a Benjamini-Hochberg–adjusted *P* < 0.05 (Supplemental Figure 5B and Supplemental Data File 2) and found that genes related to neutrophil degranulation and cytokine signaling were enriched in patients with any bacterial LRTI (Supplemental Figure 5C and Supplemental Data File 3). These results suggest the potential for developing in future work a rule-out classifier for bacterial infection that could be used to limit unnecessary antibiotic usage.

### Classification of LRTI status based on integration of host and microbial features.

**Figure 4:**
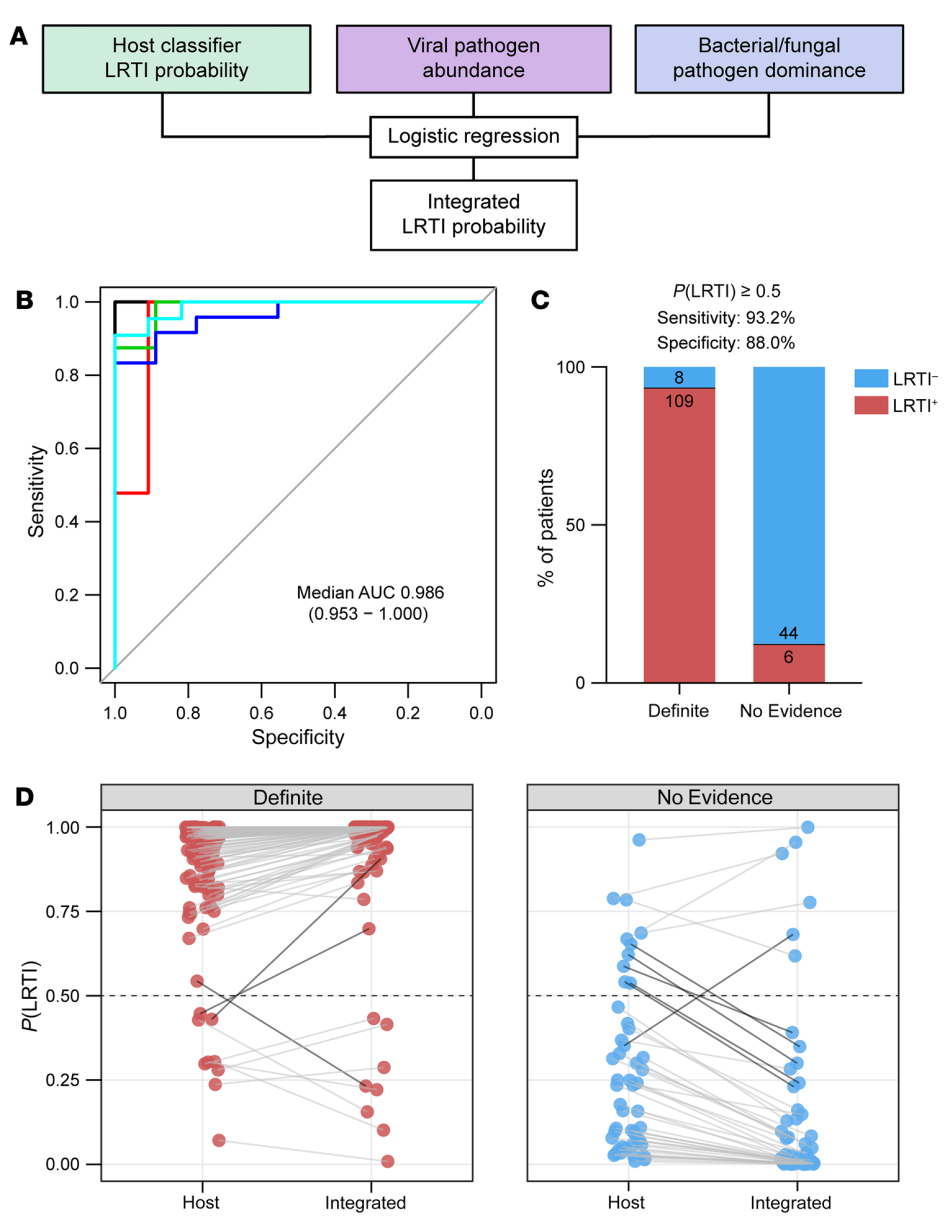
Integrated host/microbe classifier for LRTI diagnosis. (**A**) Schematic of the integrated host/microbe classifier. (**B**) Receiver operating characteristic (ROC) curve of the integrated classifier in each of the test folds. The median and range of the area under the curve (AUC) are indicated. (**C**) Bar plot showing the number and percentage of patients in the Definite and No Evidence groups who were classified according to their clinical adjudication using a 50% out-of-fold probability threshold. (**D**) The shift in out-of-fold LRTI probability from the host classifier to the integrated classifier for patients in the Definite (left) and No Evidence (right) groups. Dark connecting lines highlight patients whose LRTI probability shifted across the 50% threshold.

Next, we asked whether integrating the host and microbial features could improve the performance of metagenomic LRTI classification. We fit a logistic regression model on the following features: (a) the LRTI probability output of the host classifier, (b) the summed abundance, measured in rpM, of any pathogenic viruses present after background filtering (the viral score), and (c) the proportion of the potentially pathogenic bacteria/fungi identified by the RBM out of all nonhost read counts (the bacterial score) (Figure 4A). As expected, the host and microbial features were correlated across most samples, but some notable exceptions were observed (Supplemental Figure 6).

The integrated classifier achieved a median AUC of 0.986 (range, 0.953–1.000) when assessed by 5-fold cross-validation (Figure 4B and Supplemental Table 5), applying the same train/test splits from the host-only cross-validation. Using a 50% out-of-fold probability threshold, the integrated classifier assigned 109 of 117 (93%) patients in the Definite group as LRTI^+^ and 44 of 50 (88%) patients in the No Evidence group as LRTI^–^ (Figure 4C and Supplemental Table 6). Compared with the host-only classifier, a net of 5 additional patients were now classified according to their clinical adjudication, and the confidence of patient classifications increased, as reflected by more extreme output probabilities (Figure 4D). Reassuringly, all patients in the No Evidence group with a diagnosis of nonpulmonary sepsis (*n* = 6) were classified as LRTI^–^, despite suffering an infection elsewhere in the body (Supplemental Table 7). We note that at a 15% out-of-fold probability threshold, the integrated classifier’s sensitivity for LRTI in the Definite group rose to more than 98%, suggesting a use case as a rule-out test for LRTI.

**Figure 5:**
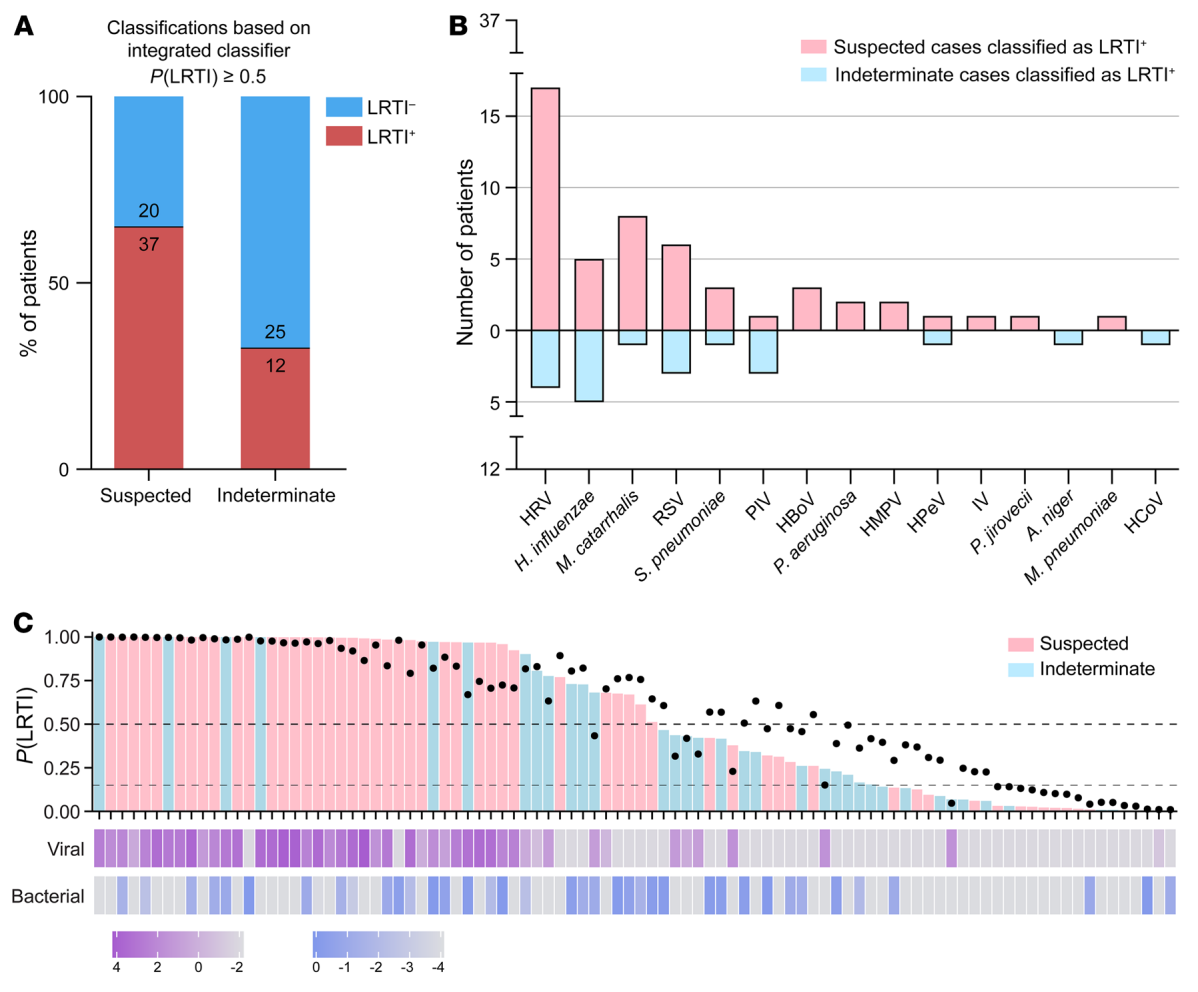
Application of the integrated classifier to patients in the Suspected and Indeterminate groups. (**A**) Bar plot showing the number and percentage of patients in the Suspected and Indeterminate groups who were classified as LRTI^+^ by the integrated classifier using a 50% probability threshold. (**B**) Viruses detected by mNGS and bacteria/fungi identified by the rules-based model (RBM) across the patients classified as LRTI^+^ in the Suspected and Indeterminate groups. HRV, human rhinovirus; RSV, respiratory syncytial virus; PIV, parainfluenza virus; HBoV, human bocavirus; HMPV, human metapneumovirus; HPeV, human parechovirus; IV, influenza virus; HCoV, human coronavirus. (**C**) Overview of inputs and output of the integrated classifier for all patients in the Suspected and Indeterminate groups. Top bars denote the integrated probability of LRTI and are colored by patient group; black dots represent the input host LRTI probability; bottom vertical bars show the input log_10_-transformed viral and bacterial scores. Dashed lines indicate the 50% LRTI probability threshold and the 15% rule-out threshold.

Finally, we trained the integrated host/microbe classifier on all the patients in the Definite and No Evidence groups and then applied it to the patients in the Suspected and Indeterminate groups, whose clinical diagnosis was less certain. The integrated classifier indicated that 37 of 57 (65%) patients in the Suspected group were LRTI^+^ compared with 12 of 37 (32%) patients in the Indeterminate group (Figure 5A), consistent with the stronger clinical suspicion of LRTI in the former case. Across all 49 patients classified as LRTI^+^ in these groups, likely pathogens (viral, bacterial, or fungal) were identified in 48 patients (98%). Pathogens detected included common (e.g., rhinovirus, *H*. *influenzae*), uncommon (e.g., bocavirus, parechovirus), and difficult to culture (e.g., *Mycoplasma pneumoniae*) microbes (Figure 5B). We also designed a visual summary incorporating all 3 inputs of the integrated classifier and its output LRTI probability (Figure 5C).

## Discussion

LRTI involves a dynamic relationship among pathogen, lung microbiome, and host response that is not captured by existing clinical diagnostic tests. Here, we demonstrated that mNGS of lower respiratory samples enables accurate LRTI diagnosis based on features of each of these key elements in critically ill children, a demographic facing a high burden of LRTI. We built on proof-of-concept work in adults (22) to develop the first, to our knowledge, fully integrated host/microbe LRTI diagnostic classifier and validated its performance in a large, multicenter prospective cohort.

Incidental carriage of pathogens in the respiratory tract is common in children (3, 5, 9–12). Consistent with this, detection of a pathogen by mNGS was in many cases insufficient for accurate LRTI diagnosis in our cohort. Among patients in the No Evidence group, 40% had potentially pathogenic microbes detected by mNGS even after application of the RBM (for bacteria and fungi). This is notably different from adults, for whom both clinical and metagenomic studies have demonstrated much lower rates of incidental pathogen carriage (7, 22). Profiling the host response is, thus, particularly important for pediatric LRTI diagnosis, as it provides evidence of an immune response to infection.

Remarkably, an LRTI diagnostic classifier based on host gene expression performed very well on its own, with a median AUC of 0.967 by cross-validation. The host signature was driven by activation markers of T cells, alveolar macrophages, and the interferon response and successfully captured cases of viral infection, bacterial infection, or coinfection. This performance suggests the gene signature could be incorporated into a clinical PCR assay as a standalone rapid diagnostic. It is likely that an even more parsimonious signature than the one used in the mNGS classifier would suffice, as 6 genes exhibited the most discriminating power.

The integrated host/microbe classifier achieved a median AUC of 0.986 by cross-validation. The incorporation of microbial features increased the confidence of LRTI classification, even though relatively few patients switched their assigned diagnosis. It is likely that the integrated classification approach will prove even more valuable in settings where the host signature may not perform as well on its own (e.g., immune-compromised patients) and will generalize better to future cohorts. Moreover, it provides clinicians with a unified framework both for LRTI diagnosis and etiologic pathogen identification.

Unlike for host gene expression, the microbial features in the integrated classifier were not automatically selected by training on identified taxa and their features in the Definite and No Evidence groups. Such an approach was not feasible given the sparse presence of individual respiratory pathogens across patients in the cohort, especially in the No Evidence group. As larger data sets are generated, it may be possible to use machine-learning approaches to capture the “null distribution” of incidentally carried pathogens in the lower respiratory tract and identify outlier cases that signal LRTI. Even then, designating a specific microbe as a “true” causal pathogen for training purposes would be nontrivial, especially in cases of coinfection. Instead, we defined summary viral and bacterial scores motivated by accumulated clinical and microbiologic knowledge. For bacteria and fungi, we took advantage of the collapse of lung microbiome diversity in the setting of pathogen dominance, an established feature of LRTI (22, 34, 35).

Comparison of mNGS and clinical microbiologic testing was complicated by inherent differences in the anatomical site of testing (upper respiratory viral PCR vs. lower respiratory mNGS) or the question addressed (growth in culture vs. dominance by mNGS) as well as by heterogeneity in microbiologic practices among study sites. Nevertheless, when clinical testing identified a microbe, it was in most cases present in the mNGS data. A notable exception was adenovirus, which was consistently not found by mNGS when detected by NP swab PCR. This could reflect sensitivity limitations of RNA-Seq for a DNA virus or true absence in the lower airway. Our secondary analysis revealing higher concordance of PCR and mNGS when performed on the same lower respiratory specimens, however, argues for the latter possibility. Future work could examine targeted enrichment strategies (37, 38) to improve detection of this or any other pathogen that proves challenging to capture by mNGS. Regardless, our findings highlight the value of concomitant assessment of the host response, which can accurately inform LRTI status even when pathogens are not detected.

A key advantage of mNGS is the capacity to provide a microbiologic diagnosis when traditional clinical testing returns negative, as in an estimated 20%–60% of suspected community- or hospital-acquired pneumonia cases (3, 6–8). Indeed, the integrated mNGS classifier confirmed LRTI in 65% of children with suspected infection but negative clinician-ordered testing in our cohort and in 32% of patients with respiratory failure of indeterminate etiology. It also provided a microbiologic diagnosis in all but one of these patients, highlighting the potential to inform pathogen-targeted versus empirical treatment.

Acute respiratory illnesses are a leading contributor to inappropriate antimicrobial use, a practice driven by challenges in distinguishing LRTI from noninfectious causes of respiratory failure or distinguishing bacterial from viral LRTI. Reflecting this is the observation that 90% of children in our cohort received empirical antimicrobials by the time of sample collection, including 84% in the No Evidence group. Host/microbe mNGS offers an opportunity for improved antimicrobial stewardship, particularly in clinically uncertain cases, by providing a probability of infection and by nominating the likely pathogen. In fact, we found that the integrated classifier could be tuned to achieve more than 98% sensitivity for LRTI detection, highlighting its potential use as a rule-out test to help exclude the need for antimicrobials. Moreover, our host gene expression analysis revealed potential for development of a host classifier specifically for bacterial infection.

Our study has several limitations that should be kept in mind. In developing the mNGS classifiers, we relied on retrospective clinical adjudication for designating the “ground truth” LRTI status of patients in the cohort. Retrospective adjudication, which considers the context of patient trajectory and clinical data not available at the time of initial admission, was the only practical approach. However, by nature, it is not infallible and was subject to variability in clinical and microbiologic practices across study sites and to the known limitations of standard microbiologic diagnostics. Moreover, comprehensive microbiologic testing was not always performed in the No Evidence group in the absence of clinical suspicion of LRTI, which likely allowed a few patients into this group who were suffering from unrecognized infection on top of their primary diagnosis. It is thus likely that some patients in the No Evidence group deemed LRTI^+^ by the mNGS classifier were not truly misclassified, but rather incorrectly adjudicated. Study limitations also include the different age distributions of comparator groups and the relative paucity of purely bacterial infections.

mNGS provides a broad screen for bacteria, viruses, and other pathogens to overcome the limitations of traditional clinical microbiologic tests. Assays utilizing this technique are already in use in hospitals for microbe detection in typically sterile compartments, such as blood (sepsis) and cerebrospinal fluid (meningitis), with turnaround within 48 hours (39, 40). mNGS promises to improve the diagnosis and treatment of respiratory infections as well (9, 22, 41–45) but has not yet seen clinical translation in this area. Respiratory samples present a special challenge because they harbor microbial communities, including potential pathogens, even in states of health. Host gene expression can help distinguish bona fide infection, and several studies have demonstrated the utility of blood transcriptional profiling for this purpose (16, 17, 20). However, this approach precludes identification of the etiologic respiratory pathogens. Simultaneous analysis of host and microbe in respiratory samples informs both questions, and it is increasingly being applied in studies of the upper and lower airway (22, 46–48). Our work now provides the first, to our knowledge, fully integrated host/microbe LRTI diagnostic classifier from lower airway mNGS, applicable across pathogen types, thus setting the stage for clinical implementation in the relatively near future.

We envision the approach for LRTI diagnosis by lower airway host/microbe mNGS outlined in this study being used at the time of intubation for critically ill children with acute respiratory failure, as a complement to traditional culture and PCR-based microbiologic testing. Our approach would need to be independently validated and its effect on clinical outcomes would need to be evaluated in a randomized clinical trial before deployment in the hospital. Future work should also examine the trajectory of patient LRTI classification over time, as infection resolves, and how well the classifier might generalize to a similarly large and heterogeneous adult cohort.

## Methods

### Study cohort.

We conducted a secondary analysis of a prospective cohort study of mechanically ventilated children admitted to 8 pediatric intensive care units in the National Institute of Child Health and Human Development’s Collaborative Pediatric Critical Care Research Network (CPCCRN) from February 2015 to December 2017 (9, 25).

We enrolled children aged 31 days to 18 years who were expected to require mechanical ventilation via endotracheal tube for at least 72 hours. Exclusion criteria included inability to obtain a TA sample from the patient within 24 hours of intubation; presence of a tracheostomy tube or plans to place one; any condition in which deep tracheal suctioning was contraindicated; a previous episode of mechanical ventilation during the hospitalization; family/team lack of commitment to aggressive intensive care as indicated by “do-not-resuscitate” orders and/or other limitation of care; or previous enrollment into this study. Some patients were ultimately excluded from the present analysis based on sequencing metrics, as described in the following.

Parents or other legal guardians of eligible patients were approached for consent by study-trained staff as soon as possible after intubation. Waiver of consent was granted for TA samples to be obtained from standard-of-care suctioning of the endotracheal tube until the parents or guardians could be approached for informed consent.

Prospectively collected clinical data were recorded in a web-based research database maintained by the CPCCRN data-coordinating center at the University of Utah, Salt Lake City, Utah, USA.

### Clinical microbiologic diagnostics.

Enrolled patients received standard-of-care clinical respiratory microbiologic diagnostics, as ordered by treating clinicians at each study site. These diagnostics included NP swab respiratory viral testing by multiplex PCR and/or TA bacterial and fungal semiquantitative cultures. Clinical diagnostic tests on samples obtained within 48 hours of intubation were included in the analyses. Microbes reported by the clinical laboratory as representing laboratory, skin, or environmental contaminants, or reported as mixed upper respiratory flora, were excluded.

### Adjudication of LRTI status.

Adjudication of LRTI status was performed without knowledge of the mNGS results and depended on the combination of two elements: (a) a retrospective clinical diagnosis made by study-site clinicians, who reviewed all clinical, laboratory, and imaging data available at the end of the admission, and (b) any standard-of-care respiratory microbiologic diagnostics (NP swab viral PCR and/or TA culture) performed on specimens collected during the first 48 hours of intubation. Patients were assigned to their LRTI status group as follows: (a) Definite, if clinicians made a diagnosis of LRTI and the patient had clinical microbiologic findings; (b) Suspected, if clinicians made a diagnosis of LRTI, but there were no microbiologic findings; (c) Indeterminate, if no diagnosis of LRTI was made despite some microbiologic findings; and (d) No Evidence, if clinicians identified a clear noninfectious cause of acute respiratory failure and no clinical or microbiologic suspicion of LRTI arose. We note that comprehensive microbiologic testing was not always performed in the No Evidence group in the absence of clinical suspicion.

### Sample collection, processing, and mNGS.

TA was collected within 24 hours of intubation, mixed 1:1 with DNA/RNA Shield (Zymo), and frozen at –80°C. RNA was extracted from 300 μL patient TA using bead-based lysis and the Allprep DNA/RNA kit (Qiagen), which included a DNase treatment step. RNA was reverse transcribed to generate cDNA, and sequencing library preparation was performed using the NEBNext Ultra II Library Prep Kit (New England BioLabs). RNA-Seq libraries underwent 150 bp paired-end sequencing on an Illumina Novaseq 6000 instrument.

### Host gene expression analysis.

Following demultiplexing, sequencing reads were pseudo-aligned with kallisto (49) (including bias correction) to an index consisting of all transcripts associated with human protein coding and long noncoding RNA genes (ENSEMBL v.99). We excluded samples with less than 500,000 estimated counts associated with transcripts of protein-coding genes. Gene-level counts were generated from the transcript-level abundance estimates using the R package tximport (50), with the scaledTPM method.

Genes were retained for differential expression (DE) analysis if they had at least 10 counts in at least 20% of the samples included in the analysis. DE analyses were performed with the R package limma (51), using quantile normalization and the voom method. *P* values were calculated using moderated *t* tests (2 tailed), as implemented in limma, and adjusted for multiple hypothesis testing with the Benjamini-Hochberg method. Tests with *P* < 0.05 were considered significant. Full DE results comparing (a) patients in the Definite and No Evidence groups and (b) patients in the Definite group with any bacterial LRTI and with purely viral LRTI are provided as Supplemental Data File 2.

Gene set enrichment analyses (52) were performed using the fgseaMultilevel function in the R package fgsea (53), which calculates pathway *P* values using an adaptive, multilevel splitting Monte Carlo approach. The analysis was applied to REACTOME (54) pathways with a minimum size of 10 genes and a maximum size of 1,500 genes. All genes from the respective DE analysis were included as input, preranked by the DE test statistic. The gene sets shown in the figures were manually selected to reduce redundancy and highlight diverse biological functions from among those with a Benjamini-Hochberg–adjusted *P* < 0.05. Full gene set enrichment analyses results are provided as Supplemental Data File 3.

### Classification of LRTI status based on host gene expression features.

Genes with at least 10 counts in at least 20% of the patients in the Definite (*n* = 117) and No Evidence (*n* = 50) groups were used as input for the host-based LRTI classification (*n* = 13,323). We applied a variance-stabilizing transformation to the gene counts, as implemented in the R package DESeq2 (55).

We implemented a 5-fold cross-validation procedure, such that in each train/test split, we (a) used LASSO logistic regression on the samples in the training folds for feature (gene) selection, (b) trained a random forest classifier on the samples in the training folds using only the selected features, and (c) applied the random forest classifier to the samples in the test fold to obtain an out-of-fold host probability of LRTI. We required at least 9 patients from the No Evidence group in each of the folds to ensure sufficient negative samples in each test set.

Simple LASSO logistic regression was fit using the cv.glmnet(family=’binomial’) function from the R package glmnet (56), leaving all other parameters at their defaults. We used the 1se criterion for selecting the tuning parameter, which picks the sparsest value of the tuning parameter that lies within 1 standard error of the optimum. When evaluating test error, we selected the tuning parameter via nested cross-validation within the training set only.

Random forest was implemented using the R package randomForest (57). We used 10,000 trees and left all parameters at their defaults.

The AUC for each test fold was calculated using the R package pROC (58) with default behavior. Sensitivity and specificity were calculated using a predetermined 50% out-of-fold LRTI probability threshold.

### Detection of microbes by mNGS and background filtering.

We processed patient TA samples alongside water controls through the open-source CZ-ID (formerly called IDSeq) metagenomic analysis pipeline (59). The pipeline performs subtractive alignment of the human genome and then reference-based alignment of the remaining reads at both the nucleotide and amino acid level against sequences in the National Center for Biotechnology Information (NCBI) nucleotide and nonredundant databases, respectively. This is followed by assembly of the reads matching each taxon. Taxa with ≥5 read counts in the nucleotide alignment and an average assembly nucleotide alignment ≥70 bp were retained for downstream analysis.

Water controls enabled estimation of the number of background reads expected for each taxon, as previously described (9, 47). This was done by modeling the number of background reads as a negative binomial distribution with mean and dispersion fitted on the water controls. For each batch (sequencing run) and taxon, we estimated the mean parameter of the negative binomial distribution by averaging the read counts across the water controls after normalizing by the total nonhost reads, slightly regularizing this estimate by including the global average (across all batches) as an additional sample. We estimated a single dispersion parameter across all taxa and batches using the functions glm.nb() and theta.md() from the R package MASS (60). Taxa were then tested for whether they exceeded the count expected from the background distribution, and a Benjamini-Hochberg adjustment was applied to all tests performed in the same sample. Taxa were considered present in a sample if they achieved an adjusted *P* < 0.05.

Any virus with known ability to cause LRTI, based on a previously conducted literature curation (22), that was present in a patient sample after background filtering was considered a probable pathogen.

### RBM.

For bacteria and fungi that were present after background filtering, we additionally applied the RBM to distinguish potential pathogens from likely commensals (22). To apply the RBM on each sample, we (a) retained the most abundant bacterial/fungal species from each genus and any less abundant species in the genus that had known ability to cause LRTI, based on a previously conducted literature curation (22); (b) ranked all the retained species in the sample by abundance and limited, at most, to the top 15; (c) identified the largest drop in abundance between the ranked species in the sample; and (d) deemed any species above the largest drop in abundance with known ability to cause LRTI as a potential pathogen.

### Analysis of microbiome diversity.

The Shannon diversity index was calculated using either all viral and bacterial taxa, or only bacterial taxa, that were present after background filtering using the R package Vegan (61). Two-sided Mann-Whitney tests with Bonferroni’s correction were used to evaluate statistical significance of group differences. Tests with *P* < 0.05 were considered significant.

### Classification of LRTI status based on integration of host and microbial features.

For the integrated host/microbe LRTI classifier, we fit a logistic regression model on the following underlying features: (a) the host LRTI probability; (b) the summed abundance, measured in rpM, of any pathogenic viruses present after background filtering (the viral score); and (c) the proportion of any potentially pathogenic bacteria/fungi identified by the RBM of all nonhost read counts (the bacterial score).

To avoid any leakage from the test set affecting the host probabilities of the training samples in the context of cross-validation, we always used the out-of-bag “votes” generated when fitting the host random forest classifier on the training set as the host probabilities of the training samples in the integrated classifier, rather than using their out-of-fold host probabilities. We applied a logistic (log-odds) transformation to the host probabilities:

 (Equation 1)
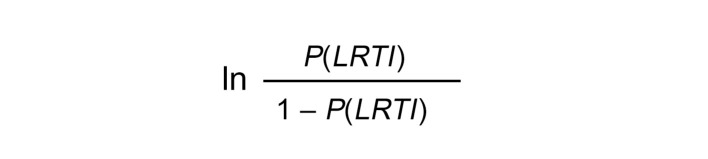


To facilitate the transformation, we first slightly regularized the host probabilities and their complementary probabilities away from 0 and 1 by a quantity inversely proportional to the number of random forest trees (*RF_trees_* = 10,000):

 (Equation 2)
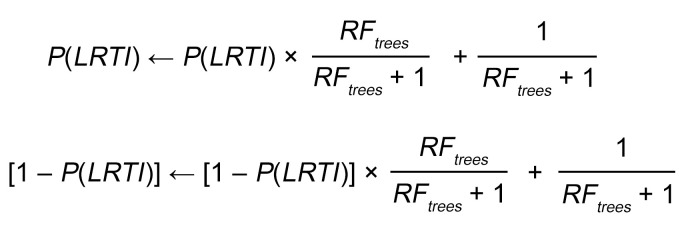


For the viral/bacterial scores, we applied a log_10_ transformation. In order to avoid taking the log of 0, we added a small uniform quantity to the scores of all the samples, which was calculated by taking the minimum non-zero viral or bacterial score, respectively, in the corresponding training set and dividing it by 10.

Performance of the integrated classifier was evaluated on the patients in the Definite and No Evidence groups using 5-fold cross-validation, with the same train/test splits and the same per-split host classifiers as in the host-only cross-validation. The AUC for each test fold was calculated using the R package pROC (58) with default behavior. Sensitivity and specificity were calculated using a predetermined 50% out-of-fold LRTI probability threshold.

The integrated classifier was then trained on all the patients in the Definite and No Evidence groups and applied to the patients in the Suspected and Indeterminate groups.

### Data and code availability.

Raw FASTQ files are protected due to patient privacy concerns. FASTQ files containing nonhost reads generated by the CZ-ID pipeline, following subtraction of reads aligning to the human genome, are available in the NCBI Sequence Read Archive database under BioProject accession PRJNA875913. All data, code, and results related to the mNGS classifier, including the host gene counts and microbial taxon counts, are available at https://github.com/eranmick/pediatric-mNGS-LRTI-classifier (main branch, commit ID6138ed0). The host gene counts are also available in the NCBI Gene Expression Omnibus database under accession GSE212532. The raw microbial data are available for interactive browsing on the CZ-ID portal under project pediatric-mNGS-LRTI-classifier (https://czid.org/pub/wmFz4U7huT).

### Statistics.

This study implemented a 5-fold cross-validation scheme to develop and evaluate performance of a binary classifier using samples with presumed known labels. Algorithms used in the classification procedure included logistic regression and random forest, which generate a probabilistic classification output. The AUC, as well as sensitivity and specificity at a predetermined probability threshold of 50%, were used as performance metrics. Statistical tests used throughout the study included moderated *t* tests (2 tailed), the Mann-Whitney test, and Fisher’s exact test, as described in detail in the corresponding sections of the Methods and in the figure and table legends.

### Study approval.

The original cohort study was approved by the Collaborative Pediatric Critical Care Research IRB at the University of Utah (protocol no. 00088656). Informed consent was obtained from parents or other legal guardians, which included permission for collected specimens and data to be used in future studies.

## Author contributions

EM, AT, JK, KLK, JLD, PMM, and CRL contributed to study conception and overall design. SC, AL, MT, AMD, NN, and CRL oversaw or performed sample processing, library preparation, and sequencing. EM, AT, JK, CMO, KMW, VS, ABM, BDW, LA, and CRL oversaw or performed collation and annotation of patient metadata. CMO, ABM, TCC, PMM, and CRL contributed to patient clinical adjudication. JK designed the underlying cross-validation scheme. EM, AT, JK, and CRL performed all data analyses and data visualizations. KLK contributed to implementation of the RBM. LA contributed to project administration and coordination. EM, AT, PMM, and CRL wrote the manuscript with input from all authors. ML, ABM, EAFS, TCC, and BDW provided advice and feedback throughout the study. PMM and CRL jointly supervised the study. EM, AT, and JK are listed as co–first authors due to equal contribution to the study, with the order of appearance determined alphabetically by last name, except in the case of JK who had departed the group by the time of manuscript preparation.

## Supplementary Material

Supplemental data

ICMJE disclosure forms

Supplemental data set 1

Supplemental data set 2

Supplemental data set 3

Supplemental data set 4

## Figures and Tables

**Table 1:**
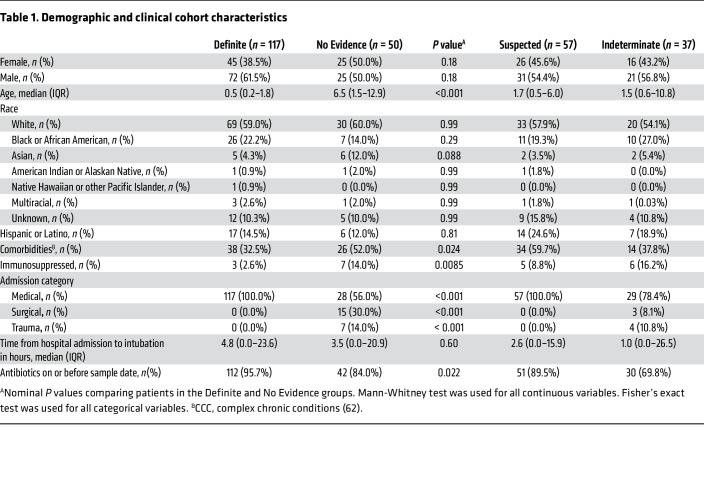
Demographic and clinical cohort characteristics

## Version 1 04/03/2023

Electronic publication
