# Supplementary material for: Integrated host/microbe metagenomics enables accurate lower respiratory tract infection diagnosis in critically ill children
Source: J Clin Invest. 2023 Apr 3;133(7):e165904. doi: 10.1172/JCI165904 (PMC10065066; doi:10.1172/JCI165904)
Supplement: Supplemental data [file jci-133-165904-s209.pdf]

Supplemental Figures

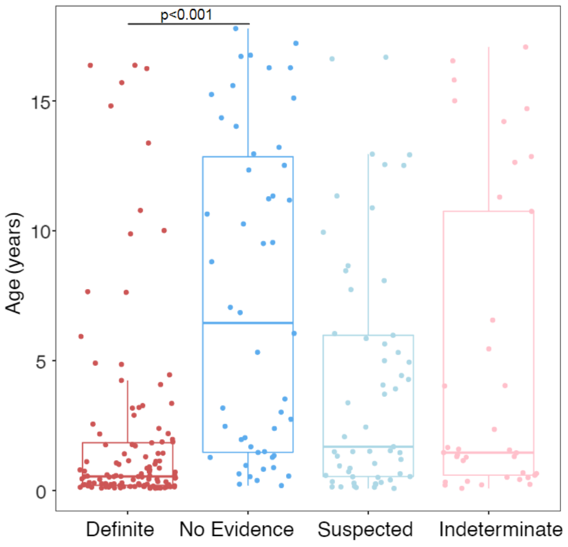

Supplemental Figure 1: Age distribution across the four LRTI status groups. P-value for the comparison between Definite and No Evidence patients was calculated using a Mann-Whitney test.

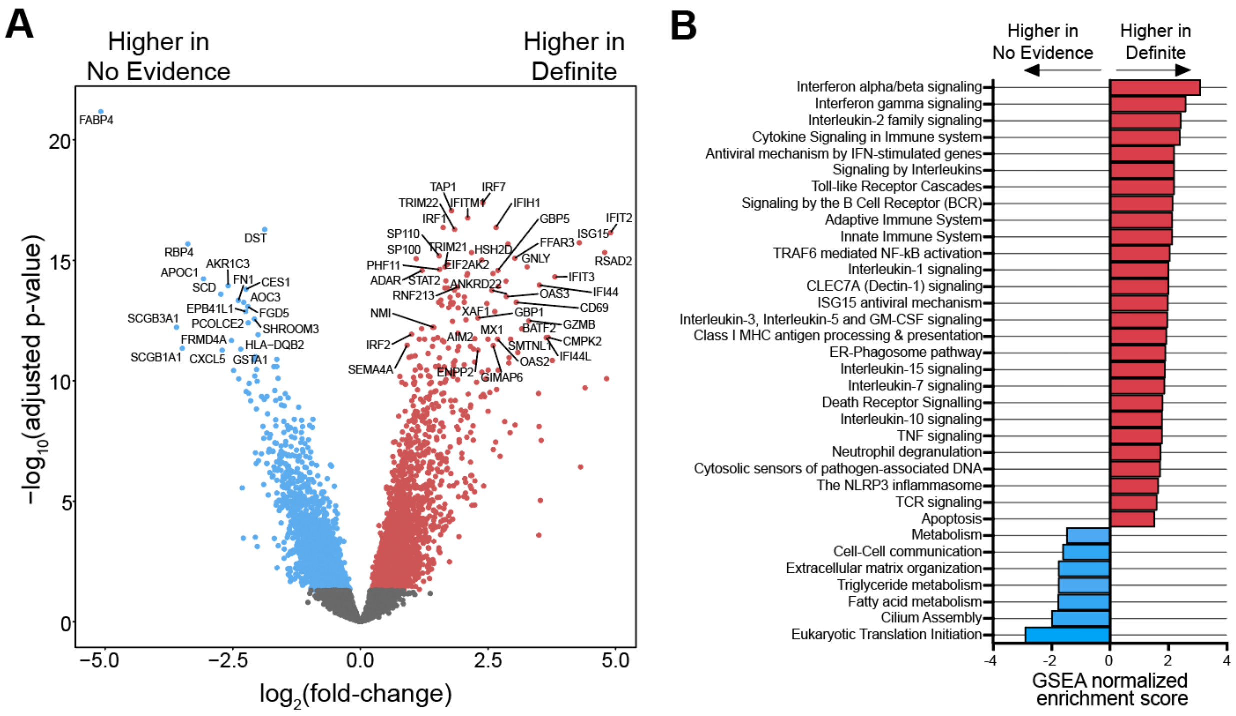

Supplemental Figure 2: A) Volcano plot highlighting genes differentially expressed (DE) between Definite and No Evidence patients. Colored genes reached statistical significance (adjusted p-value < 0.05). **B)** Normalized enrichment scores of selected REACTOME pathways that reached statistical significance (adjusted p-value < 0.05) in the GSEA based on the DE results between Definite and No Evidence patients.

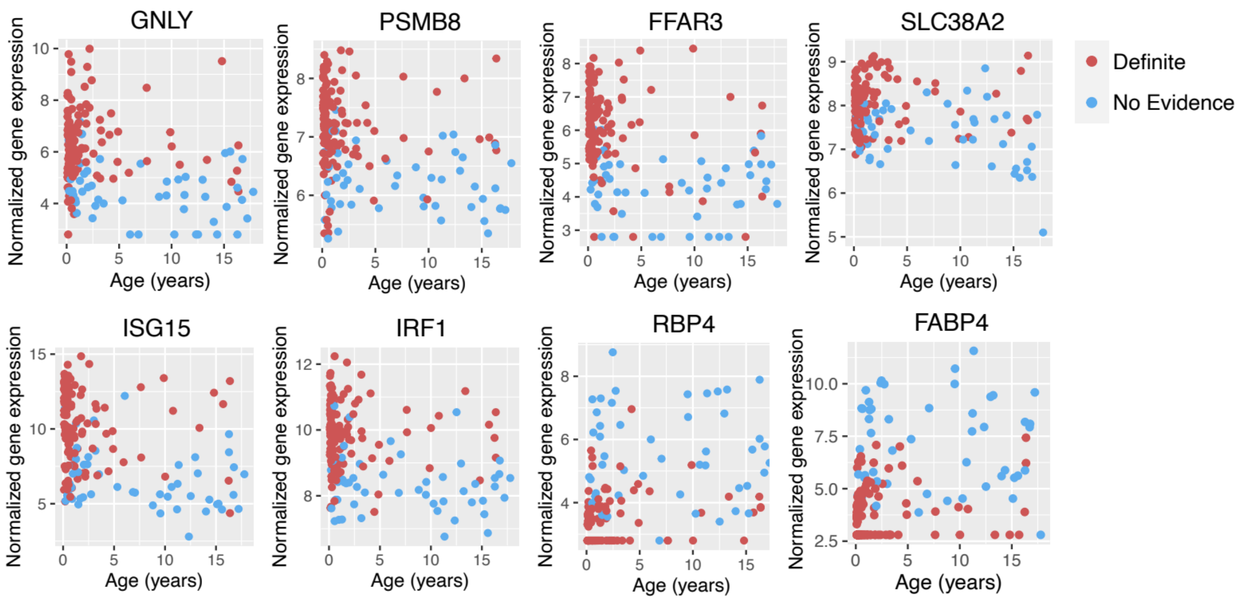

Supplemental Figure 3: Expression of eight host classifier genes as a function of age in Definite (red) and No Evidence (blue) patients.

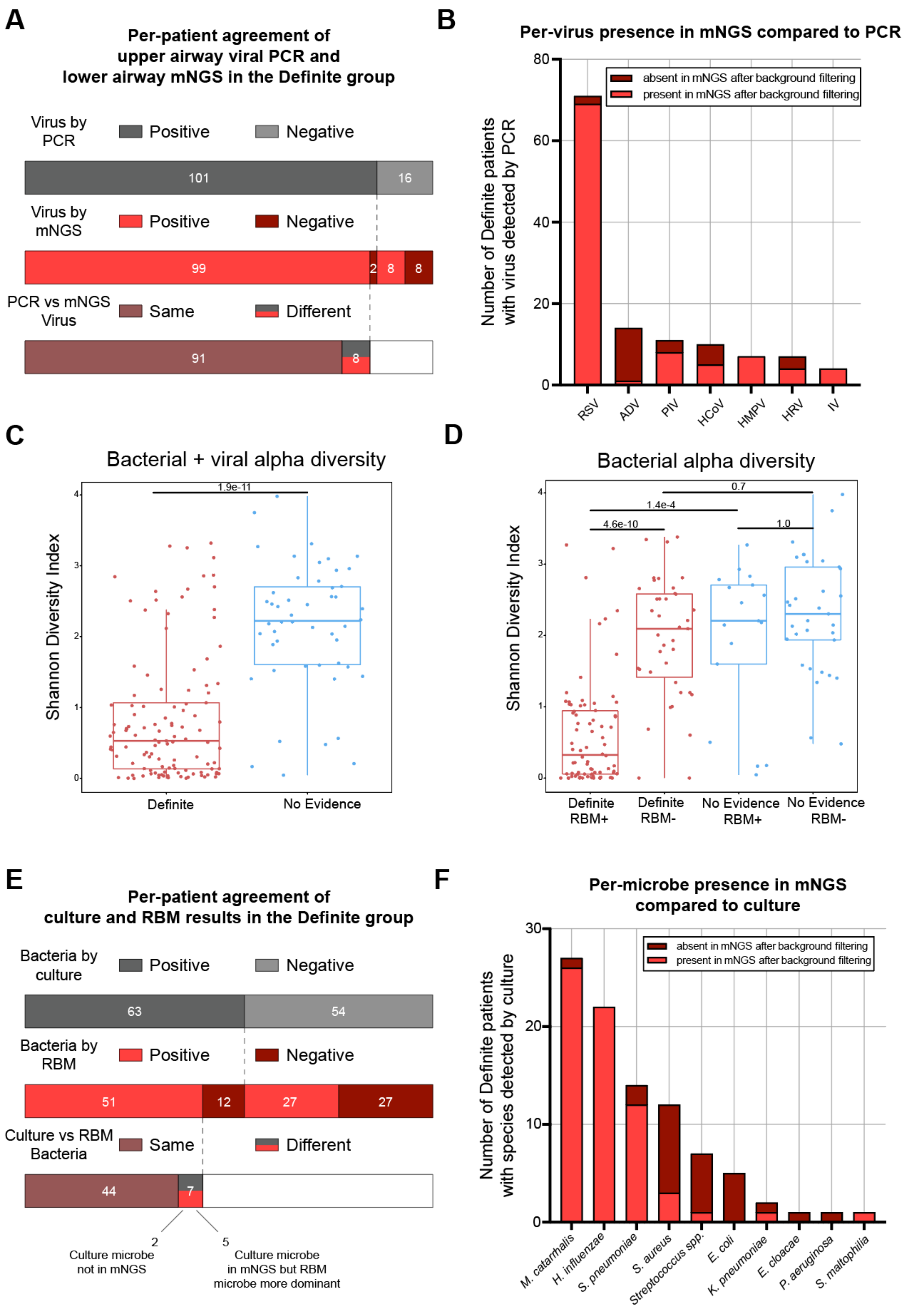
A
Per-patient agreement of
upper airway viral PCR and
lower airway mNGS in the Definite group
Virus by PCR
Positive
Negative
101
16
Virus by mNGS
Positive
Negative
99
2
8
8
PCR vs mNGS Virus
Same
Different
91
8
B
Per-virus presence in mNGS compared to PCR
absent in mNGS after background filtering
present in mNGS after background filtering
Number of Definite patients with virus detected by PCR
0
20
40
60
80
RSV
ADV
PIV
HCoV
HMPV
HRV
IV
C
Bacterial + viral alpha diversity
Shannon Diversity Index
1.9e-11
Definite
No Evidence
D
Bacterial alpha diversity
Shannon Diversity Index
0.7
1.4e-4
4.6e-10
1.0
Definite RBM+
Definite RBM-
No Evidence RBM+
No Evidence RBM-
E
Per-patient agreement of
culture and RBM results in the Definite group
Bacteria by culture
Positive
Negative
63
54
Bacteria by RBM
Positive
Negative
51
12
27
27
Culture vs RBM Bacteria
Same
Different
44
7
2
Culture microbe not in mNGS
5
Culture microbe in mNGS but RBM microbe more dominant
F
Per-microbe presence in mNGS compared to culture
absent in mNGS after background filtering
present in mNGS after background filtering
Number of Definite patients with species detected by culture
0
10
20
30
M. catarrhalis
H. influenzae
S. pneumoniae
S. aureus
Streptococcus spp.
E. coli
K. pneumoniae
E. cloacae
P. aeruginosa
S. maltophilia

Supplemental Figure 4: A) Diagram depicting the agreement at the patient level between clinical upper respiratory PCR viral testing and lower airway mNGS detection after background filtering in the Definite group. Agreement between the two methods in a patient was defined as at least one virus identified by both. **B)** Bar plot showing the number of cases of each virus detected by clinical upper respiratory PCR testing and the proportion that was also present by mNGS after background filtering. RSV, respiratory syncytial virus; ADV, adenovirus; PIV, parainfluenza virus; HCoV, human coronavirus; HMPV, human metapneumovirus; HRV, human rhinovirus; IV, influenza virus. **C)** Boxplots of bacterial+viral microbiome alpha diversity, measured by the Shannon index, in Definite and No Evidence patients. Horizontal lines denote the median, box hinges represent the interquartile range (IQR), and whiskers extend to the most extreme value no greater than 1.5*IQR from the hinges. **D)** Boxplots of bacterial-only alpha diversity measured by the Shannon index. Definite and No Evidence patients are split by whether a potential pathogen was identified by the RBM. P-values in C) and D) were calculated by a Mann-Whitney test with Bonferroni correction. **E)** Diagram depicting the agreement at the patient level between clinical culture and the results of the RBM in the Definite group. Agreement between the two methods in a patient was defined as at least one species identified by both. **F)** Bar plot showing the number of cases of each species detected by clinical culture and the proportion that was also present by mNGS after background filtering. *Streptococcus spp.*, *Streptococcus* species other than *S. pneumoniae*.

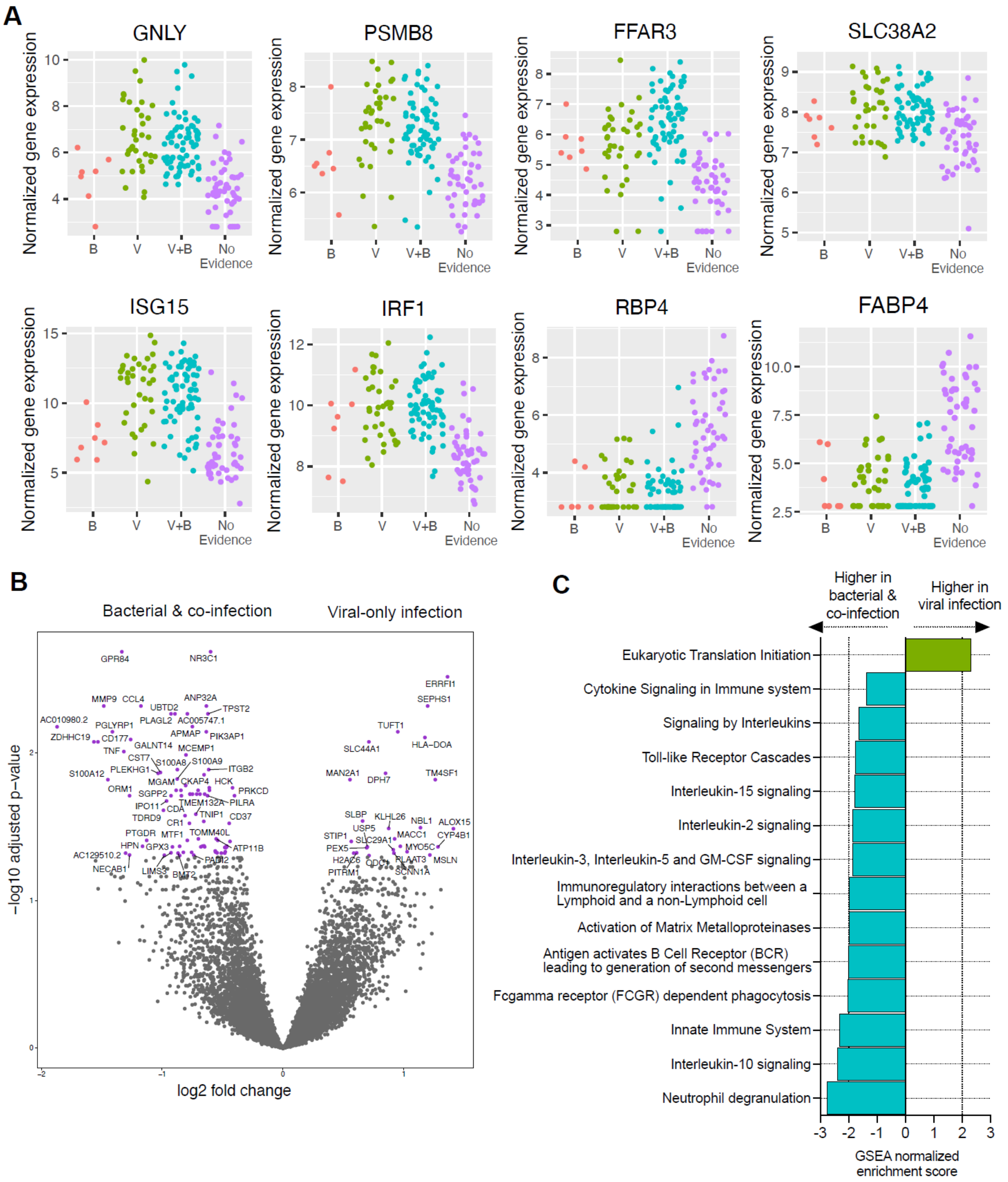

Supplemental Figure 5: A) Expression of eight host classifier genes in Definite patients with only bacterial pathogens identified by the RBM (B; n=7), only viral pathogens detected by mNGS (V; N=36), viral and bacterial pathogens (V+B; n=71), and the No Evidence patients (n=50) for comparison. Three patients from the Definite group are not shown because they did not have any pathogens identified by mNGS. One No Evidence sample in the plot of *SLC38A2* was omitted since it was an extreme outlier. **B)** Volcano plot highlighting genes differentially expressed (DE) between Definite patients with any bacterial infection (bacterial-only + co-infection) and viral-only infection. Genes colored in purple reached statistical significance (adjusted p-value < 0.05). **C)** Normalized enrichment scores of selected REACTOME pathways that reached statistical significance (adjusted p-value < 0.05) in the GSEA based on the DE results between Definite patients with any bacterial infection (bacterial-only + co-infection) and viral-only infection.

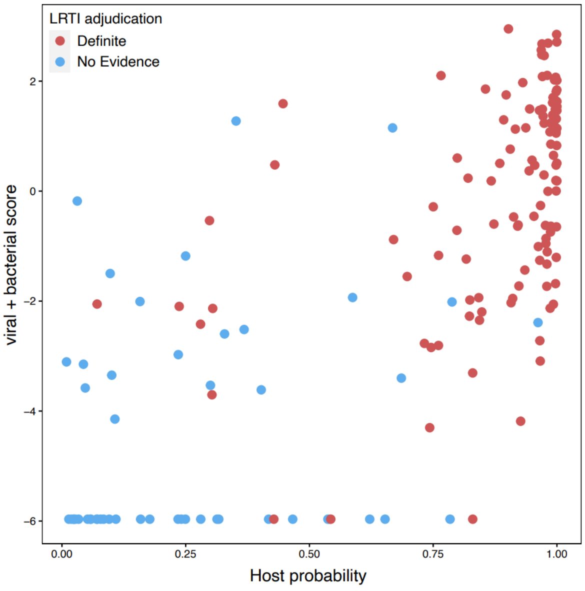

Supplemental Figure 6: Scatterplot of the host LRTI probability (x-axis) and the sum of the $\log_{10}$-transformed microbial scores (y-axis) in the Definite and No Evidence patients.

Supplemental Tables

Supplemental Table 1: Host genes selected by lasso logistic regression in each of the 5 cross-validation train/test splits, and the area under the receiver operating characteristic curve (AUC) of a random forest classifier using the selected genes.

Test fold	Gene ID	Gene symbol	Regression coefficient	AUC
1	(Intercept)	NA	-2.0280997	0.996
1	ENSG00000115523	*GNLY*	0.40435424	
1	ENSG00000114737	*CISH*	0.29736499	
1	ENSG00000185897	*FFAR3*	0.2334893	
1	ENSG00000134294	*SLC38A2*	0.23225034	
1	ENSG00000204264	*PSMB8*	0.14737796	
1	ENSG00000133101	*CCNA1*	0.13698008	
1	ENSG00000125347	*IRF1*	0.08788657	
1	ENSG00000152766	*ANKRD22*	0.01618487	
1	ENSG00000103356	*EARS2*	-0.005208	
1	ENSG00000272168	*CASC15*	-0.0129449	
1	ENSG00000163710	*PCOLCE2*	-0.0141204	
1	ENSG00000196139	*AKR1C3*	-0.0145091	
1	ENSG00000163735	*CXCL5*	-0.0202633	
1	ENSG00000164631	*ZNF12*	-0.0296153	
1	ENSG00000162929	*KIAA1841*	-0.031873	
1	ENSG00000102962	*CCL22*	-0.0470626	
1	ENSG00000196305	*IARS1*	-0.0541444	
1	ENSG00000259094	*AC013457.1*	-0.0642868	
1	ENSG00000203865	*ATP1A1-AS1*	-0.0785439	
1	ENSG00000115414	*FN1*	-0.0963792	
1	ENSG00000132300	*PTCD3*	-0.1094826	
1	ENSG00000273173	*SNURF*	-0.1236364	
1	ENSG00000138207	*RBP4*	-0.1867265	
1	ENSG00000170323	*FABP4*	-0.2260672	
1	ENSG00000182141	*ZNF708*	-0.2407744	
2	(Intercept)	NA	-0.4183313	0.953
2	ENSG00000134294	*SLC38A2*	0.21115084	
2	ENSG00000185897	*FFAR3*	0.17219249	
2	ENSG00000115523	*GNLY*	0.15221918	
2	ENSG00000204264	*PSMB8*	0.0974774	
2	ENSG00000152766	*ANKRD22*	0.06800369	
2	ENSG00000272821	*U62317.2*	0.05245633	
2	ENSG00000187608	*ISG15*	0.03953433	
2	ENSG00000162929	*KIAA1841*	-0.0224986	
2	ENSG00000196139	*AKR1C3*	-0.0991982	
2	ENSG00000170323	*FABP4*	-0.1720699	

2	ENSG00000253729	*PRKDC*	-0.2010562	
2	ENSG00000138207	*RBP4*	-0.2940594	
3	(Intercept)	NA	0.27758103	0.986
3	ENSG00000185507	*IRF7*	0.29760005	
3	ENSG00000133101	*CCNA1*	0.22848885	
3	ENSG00000115523	*GNLY*	0.20275662	
3	ENSG00000185897	*FFAR3*	0.09319602	
3	ENSG00000134294	*SLC38A2*	0.05523801	
3	ENSG00000272821	*U62317.2*	0.03959668	
3	ENSG00000196189	*SEMA4A*	0.03111383	
3	ENSG00000136231	*IGF2BP3*	0.01793655	
3	ENSG00000114737	*CISH*	0.01066616	
3	ENSG00000117143	*UAP1*	-0.0043278	
3	ENSG00000163735	*CXCL5*	-0.0087013	
3	ENSG00000113068	*PFDN1*	-0.0087115	
3	ENSG00000149021	*SCGB1A1*	-0.0088169	
3	ENSG00000232629	*HLA-DQB2*	-0.0158939	
3	ENSG00000164631	*ZNF12*	-0.0351792	
3	ENSG00000273173	*SNURF*	-0.0502438	
3	ENSG00000196139	*AKR1C3*	-0.0532949	
3	ENSG00000170324	*FRMPD2*	-0.0607793	
3	ENSG00000259094	*AC013457.1*	-0.0637096	
3	ENSG00000272660	*AC090425.2*	-0.1024448	
3	ENSG00000008226	*DLEC1*	-0.1276673	
3	ENSG00000272168	*CASC15*	-0.1499889	
3	ENSG00000170323	*FABP4*	-0.4076148	
4	(Intercept)	NA	-5.181784	0.954
4	ENSG00000175073	*VCPIP1*	0.50212823	
4	ENSG00000115523	*GNLY*	0.23413097	
4	ENSG00000135604	*STX11*	0.23226792	
4	ENSG00000168394	*TAP1*	0.13854581	
4	ENSG00000185897	*FFAR3*	0.13639215	
4	ENSG00000185885	*IFITM1*	0.07866599	
4	ENSG00000133106	*EPSTI1*	0.05899777	
4	ENSG00000158769	*F11R*	-0.0055638	
4	ENSG00000163710	*PCOLCE2*	-0.0206461	
4	ENSG00000149021	*SCGB1A1*	-0.0263191	
4	ENSG00000182141	*ZNF708*	-0.1006603	
4	ENSG00000138207	*RBP4*	-0.1413799	
4	ENSG00000170323	*FABP4*	-0.2005039	
5	(Intercept)	NA	0.5726104	0.967
5	ENSG00000204264	*PSMB8*	0.27278452	
5	ENSG00000115523	*GNLY*	0.0762971	

5	ENSG00000175073	*VCPIP1*	0.05579582	
5	ENSG00000185897	*FFAR3*	0.04184826	
5	ENSG00000188820	*CALHM6*	0.03110363	
5	ENSG00000133106	*EPSTI1*	0.02410902	
5	ENSG00000187608	*ISG15*	0.00687972	
5	ENSG00000163710	*PCOLCE2*	-0.0191247	
5	ENSG00000151914	*DST*	-0.029031	
5	ENSG00000163735	*CXCL5*	-0.1149234	
5	ENSG00000170323	*FABP4*	-0.389078	

Supplemental Table 2: Genes selected for the final host classifier by lasso logistic regression applied to all the Definite and No Evidence patients, with their regression coefficients. The number of times the gene was selected across the 5 cross-validation (CV) splits is also indicated.

Gene ID	Gene symbol	Gene product	Regression coefficient	Times selected in CV
ENSG00000115523	*GNLY*	Granulysin	0.257	5
ENSG00000204264	*PSMB8*	Proteasome subunit beta 8	0.249	3
ENSG00000185897	*FFAR3*	Free fatty acid receptor 3	0.224	5
ENSG00000134294	*SLC38A2*	Solute carrier family 38 member 2	0.214	3
ENSG00000187608	*ISG15*	ISG15 ubiquitin-like modifier	0.070	2
ENSG00000125347	*IRF1*	Interferon regulatory factor 1	0.027	1
ENSG00000162929	*KIAA1841* (also known as *SANBR*)	SANT and BTB domain regulator of class switch recombination	-0.014	2
ENSG00000272660	*AC090425.2*	Long non-coding RNA, antisense to *ACTL6A*	-0.016	1
ENSG00000196139	*AKR1C3*	Aldo-keto reductase family 1 member C3	-0.019	3
ENSG00000163735	*CXCL5*	C-X-C motif chemokine ligand 5	-0.019	3
ENSG00000080546	*SESN1*	Sestrin 1	-0.033	0
ENSG00000163710	*PCOLCE2*	Procollagen C-endopeptidase enhancer 2	-0.033	3
ENSG00000138207	*RBP4*	Retinol binding protein 4	-0.167	3
ENSG00000170323	*FABP4*	Fatty acid binding protein 4	-0.297	5
(Intercept)			-3.112	

Supplemental Table 3: Differential expression results for the 14 final classifier genes comparing: **A)** No Evidence patients under four years old (n=23; median age 1.3 years) versus over four years old (n=27; median age 12.5), and **B)** Definite patients under four years old (n=100; median age 0.4) versus No Evidence patients under four years old (n=23; median age 1.3).

A

Gene symbol	Log_2 fold-change	P-value	Adjusted P-value
GNLY	-1.14	0.01	0.72
PSMB8	-0.33	0.21	0.82
FFAR3	-0.43	0.33	0.87
SLC38A2	-0.57	0.07	0.75
ISG15	-1.68	0.01	0.72
IRF1	-0.30	0.25	0.83
KIAA1841	0.14	0.59	0.94
AC090425.2	1.48	0.07	0.76
AKR1C3	0.81	0.10	0.76
CXCL5	0.24	0.67	0.95
SESN1	0.27	0.47	0.91
PCOLCE2	0.54	0.21	0.81
RBP4	-0.04	0.95	0.99
FABP4	-0.56	0.45	0.90

B

Gene symbol	Log_2 fold-change	P-value	Adjusted P-value
GNLY	2.73	2.11E-08	1.46E-06
PSMB8	1.11	7.78E-08	4.22E-06
FFAR3	2.93	9.70E-12	4.25E-09
SLC38A2	0.60	2.50E-05	4.33E-04
ISG15	3.49	3.71E-09	3.89E-07
IRF1	1.52	5.18E-12	2.88E-09
KIAA1841	-0.89	2.84E-05	4.76E-04
AC090425.2	-0.18	7.50E-01	8.56E-01
AKR1C3	-2.48	2.24E-12	1.42E-09
CXCL5	-2.63	4.62E-09	4.52E-07
SESN1	-0.53	4.92E-02	1.38E-01
PCOLCE2	-2.06	2.60E-09	2.93E-07
RBP4	-3.64	1.51E-17	1.00E-13
FABP4	-5.54	4.02E-26	5.36E-22

Supplemental Table 4: Comparison of mNGS viral detection in TA samples with PCR viral detection in nasopharyngeal (NP) swabs or in the same TA samples in a subset of patients.

**Definite patients with matched NP swab and TA viral PCR testing (n=21)**	**Agreement of mNGS with NP swab PCR**	**Concordance of mNGS and NP swab PCR**	**Agreement of mNGS with TA PCR**	**Concordance of mNGS and TA PCR**
All viruses	22/34 = 64.7%	22/37 = 59.5%	23/24 = 95.8%	23/26 = 88.5%
Respiratory syncytial virus	11/12 = 91.7%	11/12 = 91.7%	10/10 = 100%	10/11 = 90.9%
Rhinovirus	4/7 = 57.1%	4/10 = 40%	6/6 = 100%	6/7 = 85.7%
Adenovirus	0/6 = 0%	0/6 = 0%	0/0 = 100%	0/0 = 100%
Coronavirus	2/3 = 66.7%	2/3 = 66.7%	2/2 = 100%	2/2 = 100%
Human metapneumovirus	3/3 = 100%	3/3 = 100%	3/3 = 100%	3/3 = 100%
Parainfluenza virus	2/3 = 66.7%	2/3 = 66.7%	2/3 = 66.7%	2/3 = 66.7%

Agreement reflects the number of viruses detected by mNGS out of the total number of viruses detected by PCR.
Concordance reflects the number of viruses detected by both mNGS and PCR out of the total number of viruses detected by at least one method.

Supplemental Table 5: Per-fold area under the curve (AUC) values for the integrated host/microbe logistic regression classifier.

**Test fold**	**AUC**
1	1.000
2	0.953
3	0.986
4	0.963
5	0.988

Supplemental Table 6: A) mNGS and clinical respiratory microbiology results for the Definite and No Evidence patients whose integrated LRTI classification was inconsistent with their adjudication. **B)** Primary diagnoses of the No Evidence patients whose integrated LRTI classification was inconsistent with their adjudication.

A

Patient	LRTI adjudication	Host *P*(LRTI)	Clinical respiratory microbiology results	mNGS viruses	Viral ΣrpM	mNGS RBM hits	Dominance of RBM hits	Integ. *P*(LRTI)
P2	Definite	0.30	*S. aureus*, HRV		0	*S. aureus*	0.62	0.10
P3	Definite	0.30	HRV, ADV	HRV C	2.15		0	0.22
P6	Definite	0.24	PIV, HCoV	PIV 4, HCoV NL63	87.61		0	0.29
P124	Definite	0.28	HCoV, *S. aureus*, *S. viridans*	HCoV 229E	41.58		0	0.41
P185	Definite	0.07	*S. maltophilia*, *S. pneumoniae*, *K. pneumoniae*		0	*S. maltophilia*, *S. pneumoniae*	0.75	0.01
P189	Definite	0.30	*S. aureus*	CMV	0.38	*S. aureus*	0.75	0.43
P195	Definite	0.54	*S. viridans*, *E. coli*		0		0	0.23
P218	Definite	0.43	*M. catarrhalis*		0		0	0.16
P1	No Evidence	0.67	No testing performed	HCoV NL63	44.94	*P. aeruginosa*	0.31	0.95
P30	No Evidence	0.35	No culture performed, negative PCR	HRV C	22.15	*M. catarrhalis*	0.85	0.68
P75	No Evidence	0.96	No culture performed, negative PCR	RSV	44.53		0	1.00
P166	No Evidence	0.79	No testing performed		0	*S. aureus*	0.82	0.92
P175	No Evidence	0.69	No testing performed	HCoV NL63	4.33		0	0.78
P250	No Evidence	0.78	Negative culture, no PCR performed		0		0	0.62

rpM, reads-per-million; RBM, rules-based model.

ADV, adenovirus
HCoV, human coronavirus
CMV, cytomegalovirus
HRV, human rhinovirus
PIV, parainfluenza virus
RSV, respiratory syncytial virus

B

Patient	LRTI adjudication	Primary diagnosis
P1	No Evidence	Neurological
P30	No Evidence	Trauma
P75	No Evidence	Non-infectious respiratory distress
P166	No Evidence	Ingestion (drug/toxin)
P175	No Evidence	Ingestion (drug/toxin)
P250	No Evidence	Seizures

Supplemental Table 7: mNGS results for the No Evidence patients with a primary diagnosis of non-pulmonary sepsis.

Patient	LRTI adjudication	Host *P*(LRTI)	mNGS viruses	Viral ΣrpM	mNGS RBM hits	Dominance of RBM hits	Integ. *P*(LRTI)
P34	No Evidence	0.05		0	*E. coli*	0.02	0.00
P40	No Evidence	0.62		0		0	0.20
P51	No Evidence	0.10	RSV	0.13	*M. catarrhalis*	0.22	0.01
P116	No Evidence	0.24		0		0	0.03
P178	No Evidence	0.65		0		0	0.35
P243	No Evidence	0.18		0		0	0.01

rpM, reads-per-million; RBM, rules-based model.

RSV, respiratory syncytial virus

Supplemental Data Files

Supplemental Data File 1. Basic sample metadata.

Supplemental Data File 2. Differential expression (DE) analyses between: i) Definite and No Evidence patients; ii) Definite patients with any bacterial LRTI and with purely viral LRTI.

Supplemental Data File 3. Gene set enrichment analysis (GSEA) results from the DE between: i) Definite and No Evidence patients; ii) Definite patients with any bacterial LRTI and with purely viral LRTI.

Supplemental Data File 4. Pathogens identified in Definite patients by clinical testing and by mNGS.
